# The Exploitation of Sodium Deoxycholate-Stabilized Nano-Vesicular Gel for Ameliorating the Antipsychotic Efficiency of Sulpiride

**DOI:** 10.3390/gels10040239

**Published:** 2024-03-31

**Authors:** Marwa H. Abdallah, Mona M. Shahien, Alia Alshammari, Somaia Ibrahim, Enas Haridy Ahmed, Hanan Abdelmawgoud Atia, Hemat A. Elariny

**Affiliations:** 1 Department of Pharmaceutics, College of Pharmacy, University of Ha’il, Ha’il 81442, Saudi Arabia; alia.alshammari@uoh.edu.sa; 2 Department of Pharmaceutics, Faculty of Pharmacy, Zagazig University, Zagazig 44519, Egypt; 3 Department of Pediatrics, College of Medicine, University of Ha’il, Ha’il 81442, Saudi Arabia; m.shahin@uoh.edu.sa (M.M.S.); so.bashir@uoh.edu.sa (S.I.); 4 Department of Anatomy, College of Medicine, University of Ha’il, Ha’il 81442, Saudi Arabia; e.haridy@uoh.edu.sa; 5 Department of Anatomy and Embryology, Faculty of Medicine, Ain Shams University, Cairo 11566, Egypt; 6 Department of Pharmacology and Toxicology, College of Pharmacy, University of Ha’il, Ha’il 81442, Saudi Arabia; ha.soliman@uoh.edu.sa (H.A.A.); hem.mohammed@uoh.edu.sa (H.A.E.); 7 Department of Biochemistry, Faculty of Pharmacy, Al-Azhar University, Cairo 11651, Egypt; 8 Department of Pharmacology and Toxicology, Faculty of Pharmacy, Al-Azhar University, Cairo 11651, Egypt

**Keywords:** Sulpiride, bilosomes, transdermal drug delivery system, sodium deoxycholate, anti-psychotic

## Abstract

The present study explored the effectiveness of bile-salt-based nano-vesicular carriers (bilosomes) for delivering anti-psychotic medication, Sulpiride (Su), via the skin. A response surface methodology (RSM), using a 3^3^ Box–Behnken design (BBD) in particular, was employed to develop and optimize drug-loaded bilosomal vesicles. The optimized bilosomes were assessed based on their vesicle size, entrapment efficiency (% EE), and the amount of Sulpiride released. The Sulpiride-loaded bilosomal gel was generated by incorporating the optimized Su-BLs into a hydroxypropyl methylcellulose polymer. The obtained gel was examined for its physical properties, ex vivo permeability, and in vivo pharmacokinetic performance. The optimum Su-BLs exhibited a vesicle size of 211.26 ± 10.84 nm, an encapsulation efficiency of 80.08 ± 1.88% and a drug loading capacity of 26.69 ± 0.63%. Furthermore, the use of bilosomal vesicles effectively prolonged the release of Su over a period of twelve hours. In addition, the bilosomal gel loaded with Su exhibited a three-fold increase in the rate at which Su transferred through the skin, in comparison to oral-free Sulpiride. The relative bioavailability of Su-BL gel was almost four times as high as that of the plain Su suspension and approximately two times as high as that of the Su gel. Overall, bilosomes could potentially serve as an effective technique for delivering drugs through the skin, specifically enhancing the anti-psychotic effects of Sulpiride by increasing its ability to penetrate the skin and its systemic bioavailability, with few adverse effects.

## 1. Introduction

Sulpiride (Su), belonging to the benzamine class, is an antipsychotic medication utilized for the treatment of several psychotic diseases [1]. Psychotic disorders afflict 1% of the general population, with a high prevalence (16%) among those with a family history of schizophrenia [2]. Sulpiride is an antipsychotic medication that selectively blocks central dopamine receptors [3]. Su has garnered significant attention among many anti-psychotic medications due to its non-toxic nature, fewer extrapyramidal side effects, decreased affinity for other neural receptors, and cost-effectiveness [4]. However, Su has a number of challenges that need to be overcome. It is categorized as a class IV drug in the biopharmaceutical classification system. Consequently, Su has low solubility in water and restricted permeability through the intestines [5]. Therefore, a previously reported poor oral bioavailability (20–30%) has been demonstrated [6]. Moreover, the administration of large amounts of the medication is necessary to treat patients, leading to troublesome adverse reactions such as cardiovascular effects, sleep problems, agitation, over-stimulation and minor extrapyramidal effects [7]. Furthermore, Su exhibits an absorption window in the upper gastrointestinal tract [8]. These factors contribute to the observed low effectiveness of the drug when taken orally and its unpredictable absorption into the bloodstream through the digestive system. Therefore, there is an increasing demand for the development of approaches to improve its characteristics. Several strategies have been employed to address the challenges of the oral administration of Sulpiride. These strategies include the use of solid lipid nanoparticles [7], self-micro-emulsifying carriers [9], and solid dispersions [10]. However, the outcomes of these attempts have been limited because they have focused on the problem of Sulpiride’s poor aqueous solubility. Therefore, other platforms are required to address other routes of administration, such as transdermal application, to improve the pharmacological effectiveness of the drug.

Transdermal drug delivery (TDD) is currently an area of considerable interest for both researchers and pharmaceutical manufacturers. Transdermal drug delivery has become a feasible substitute for oral administration for systemic drug delivery. Transdermal medication delivery is becoming more popular due to its many benefits compared to oral administration. From a therapeutic point of view, it mitigates variations in the levels of medicines in the bloodstream, particularly for medications with a short half-life. In addition, the enhanced bioavailability resulting from the avoidance of first-pass metabolism allows for the administration of lower dosages to achieve the desired bioavailability. Transdermal drug delivery (TDD) enables prolonged drug release by circumventing issues related to drug absorption following oral administration, such as the pH and activity of enzymes [11]. This, in turn, reduces the systemic adverse effects and enhances the safety margin of the administered drugs [12]. The convenience and patient-friendly nature of transdermal drug delivery (TDD) is attributed to aspects such as reduced dose frequency, non-invasive administration and ease of application [13]. These factors contribute to improved patient adherence, particularly in cases of extended periods of treatment, such as in the management of chronic pain [14]. Therefore, the transdermal administration of antipsychotic drugs is a dependable approach to boost medication adherence and reduce the need for numerous doses, hence improving patient compliance. However, the most superficial layer of the skin (stratum corneum) acts as an obstacle, preventing drugs from being absorbed systemically. As a result, it may restrict the amount of drugs that can be absorbed into the bloodstream when administered through the skin [15]. El-Tokhy et al. concluded that the transdermal delivery of antipsychotics demonstrated improved therapeutic outcomes compared to oral administration [16]. However, formulations based on nanotechnology have provided several benefits and greater effectiveness compared to traditional methods. Moreover, systems based on phospholipids are highly recommended for the delivery of antipsychotics, and the lipid core effectively dissolves lipophilic compounds, resulting in a high percentage of drug loading. Alnaim et al. suggested that the use of niosomal gel formulation loaded with levosulpiride, administered transdermally, could enhance the effectiveness of the drug and may serve as a viable alternative to traditional therapy [17].

Recent studies have highlighted the potential application of nano-vesicular formulations as carriers that can improve the penetration of hydrophobic and/or hydrophilic medicines via the skin. This has been supported by several studies [15,18,19]. Phospholipid-based nano-vesicular carriers have shown effectiveness in reducing the lipid barrier of the skin [20], facilitating the permeation of drugs into the skin’s deeper layers and their absorption into the systemic circulation. Bilosomes are phospholipid- and bile-salt-based deformable and flexible lipid vesicles that exhibit significant advantages over traditional vesicles (liposomes and niosomes) with respect to high stability and a simplified manufacturing process [21]. Bilosomes serve as a drug delivery system, and have several benefits including high biocompatibility and biodegradability with little toxicity, self-assembly capability, easy removal from the body, and an enhanced effectiveness and bioavailability of enclosed substances. Therefore, the use of a biocompatible bile salt, namely sodium deoxycholate (SDC), could enhance the stability of bilosomes and exceed the stability of conventional liposomes. Bile salts are a kind of bio-surfactant that enhance the bioavailability of drugs in the presence of obstacles for absorption, such as limited permeability across cell membranes or poor solubility in water [22]. In addition, bile salts greatly reduce the temperature at which lipids undergo phase transition, resulting in bilosomal vesicles that are extremely deformable and flexible at physiological temperature. The flexibility of bilosomal vesicles greatly facilitates transdermal application through enhancing penetration into the skin’s deep layers [23]. Significantly, the presence of bile salts, sodium deoxycholate (SDC), greatly improves the stability of bilosomal vesicles, in comparison to other traditional vesicles [24]. As a result, bilosomes have been used in numerous investigations to improve the transdermal administration of various medications, including niflumic acid [25], tizanidine hydrochloride [26], and lornoxicam [27].

Bilosomal gels are polymer networks that have a three-dimensional structure and are capable of absorbing significant amounts of biological fluids or water. Due to their distinctive physical characteristics, including as biocompatibility, flexibility, biodegradability, high porosity, and controlled drug release, they are intriguing tools for drug delivery applications [28].

Currently, there are no recorded studies that have evaluated the possibility of bilosomes as carriers for delivering Sulpiride. Consequently, the main objective of the current study was to use nanovesicles along with in vitro and in vivo studies to find an innovative approach for antipsychotic medications. In addition, our goal was to design appropriate nano-vesicles for transdermal administration to ameliorate the in vivo performance of Sulpiride encapsulated in bilosomes.

In this study, we created and examined a bilosomal delivery system containing Sulpiride (Su) to assess its bioavailability after being applied to the skin. Sulpiride-loaded bilosomes were created, utilizing thin film hydration technique. The Box–Behnken design (BBD) was used to optimize the bilosomal vesicles. Subsequently, the bilosomes that were optimized underwent characterization to determine their particle size, morphology, and entrapment efficiency. In our study, the in vitro drug release, ex vivo drug permeation, and pharmacokinetics activity of Su-loaded bilosomal gel were evaluated and compared with those of free drug and Sulpiride-loaded gel.

Future prospects for the emerging promising nano-vesicular systems could involve more examinations of bilosomal fate and the use of advanced technology for targeting, as this delivery method has not yet been fully explored. Additional investigations, including in vivo and preclinical studies, should be taken in consideration in order to prepare such delivery systems for competition in the pharmaceutical industry.

## 2. Results and Discussion

### 2.1. Fabrication of Bilosomes (BLs) Loaded with Sulpiride

In the present study, Sulpiride-loaded bilosomes (Su-BLs) were prepared using the thin film hydration process. A Box–Behnken design (BBD) was employed for the optimization of Sulpiride-loaded bilosomes. Fifteen formulations were created by manipulating three formulation factors: lipid concentration % (X_1_); edge activator concentration %, SDC (X_2_); and surfactant concentration % (X_3_) (Table 1). Two dependent variables, EE% (Y_1_) and vesicle size (nm) (Y_2_), were examined to determine the impact of these formulation factors. Their values were analyzed for different design models. The ANOVA examination of the quadratic model indicated that all of the formulation factors investigated had a significant statistical impact on both Y_1_ and Y_2_, as shown in Table S1. Moreover, the expected and adjusted R^2^ values exhibited a satisfactory level of consistency.

### 2.2. Influence of Formulation Factors on the Properties of Su-BLs

#### 2.2.1. The Impact on EE%

**Figure 1:**
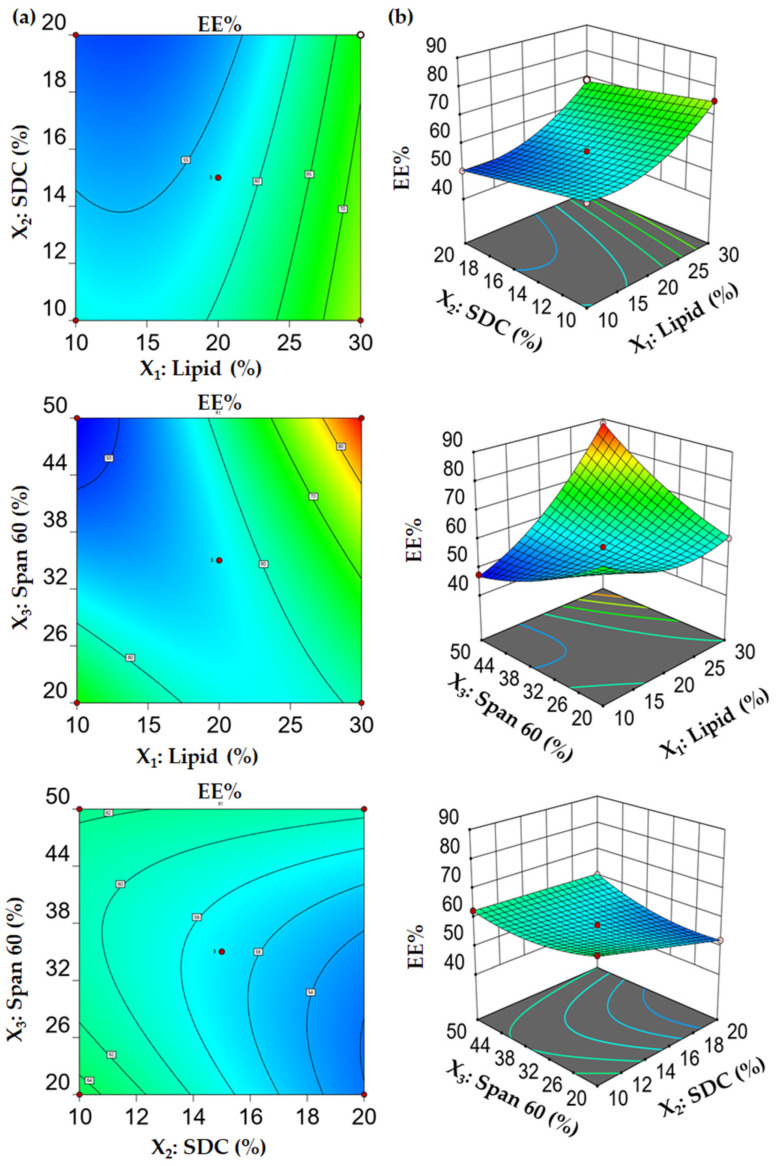
Influence of formulation factors on EE% (Y_1_). (**a**) Contour plots for Y_1_ and (**b**) 3D surface plots of Y_1_.

The potential of bilosomal vesicles to entrap a significant concentration of the pharmacological agents is a fundamental characteristic that could enhance their therapeutic usefulness. The encapsulation efficiency (EE%) of Su-BLs was influenced by several formulation factors and ranged from 47.42 ± 1.38% (F6) to 88.29 ± 3.01% (F3), as demonstrated in Table 1. Figure 1 clearly demonstrates that an increase in the lipid content led to a significant enhancement of the entrapment efficiency. When the lipid content (X_1_) was increased from 10% to 30% while keeping the SDC content (X_2_) and surfactant content (X_3_) constant, there was a notable increase in the percentage of encapsulated efficiency %. The % EE of Su-BLs (F6) containing 10% lipid, 15% SDC, and 50% Span 60 (47.42 ± 1.38%) was lower than that obtained from 30% lipid, with the same concentration of SDC and Span 60 (F3, 88.29 ± 3.01%). The observed enhancement in the percentage of encapsulated drug (Su) can be attributed to the favorable impact of lipid concentration on the surface area of the lipid bilayer. This increase in lipid concentration creates a greater area for the lipophilic medication to be trapped.

This finding aligns with the results of Abdallah et al., who emphasized the favorable impact of the concentration of the lipid on Silymarin encapsulation in transfersomes [29]. Likewise, increasing the concentration of Span 60 from 20 to 50% resulted in an improvement in the entrapment efficiency. The entrapment efficiency of F13 (52.12 ± 2.64) made with 20% of Span 60 was significantly lower than that of F4 (60.36 ± 3.07) formulated with 50% of Span 60, when the concentrations of lipid and SDC were kept constant. The elevated transition temperature of Span 60 and increased alkyl chain length (C18) may explain the better Su encapsulation efficiency in bilosomes when the concentration of Span 60 was increased [30]. Conversely, the increase in edge activator concentration (SDC) from 10% to 20% had a negative influence on the encapsulation efficiency. The efficiency of F15 prepared with 10% SDC was 65.23 ± 3.02%, which was significantly higher than the efficiency of F13 prepared with 20% SDC, which was 52.12 ± 2.64%. Studies have indicated that an increased amount of bile salts could increase the permeability of the vesicular membranes by creating openings in the membrane. This would lead to increased fluidity and the leaking of drugs [31].

The polynomial equation (1) derived from the BBD accurately corroborated our findings, demonstrating the combined impact of lipid concentration (X_1_) and the opposing effects of SDC concentration (X_2_) and surfactant concentration (X_3_) on entrapment efficiency. In order to demonstrate the model’s appropriateness and statistical significance, an ANOVA analysis was conducted. The ANOVA findings, summarized in (Table S1), indicate that the model was statistically significant, with an F value of 480.16 and a *p*-value less than 0.0001.

EE% = 56.99 + 8.53 X_1_ − 3.66 X_2_ + 1.49 X_3_ + 0.41 X_1_ X_2_ +12.30 X_1_ X_3_ + 2.75 X_2_ X_3_ + 6.18 X_1_^2^ − 0.03 X_2_^2^ + 3.08 X_3_^2^ (1)

#### 2.2.2. Impact on Vesicle Size (Y_2_)

**Figure 2:**
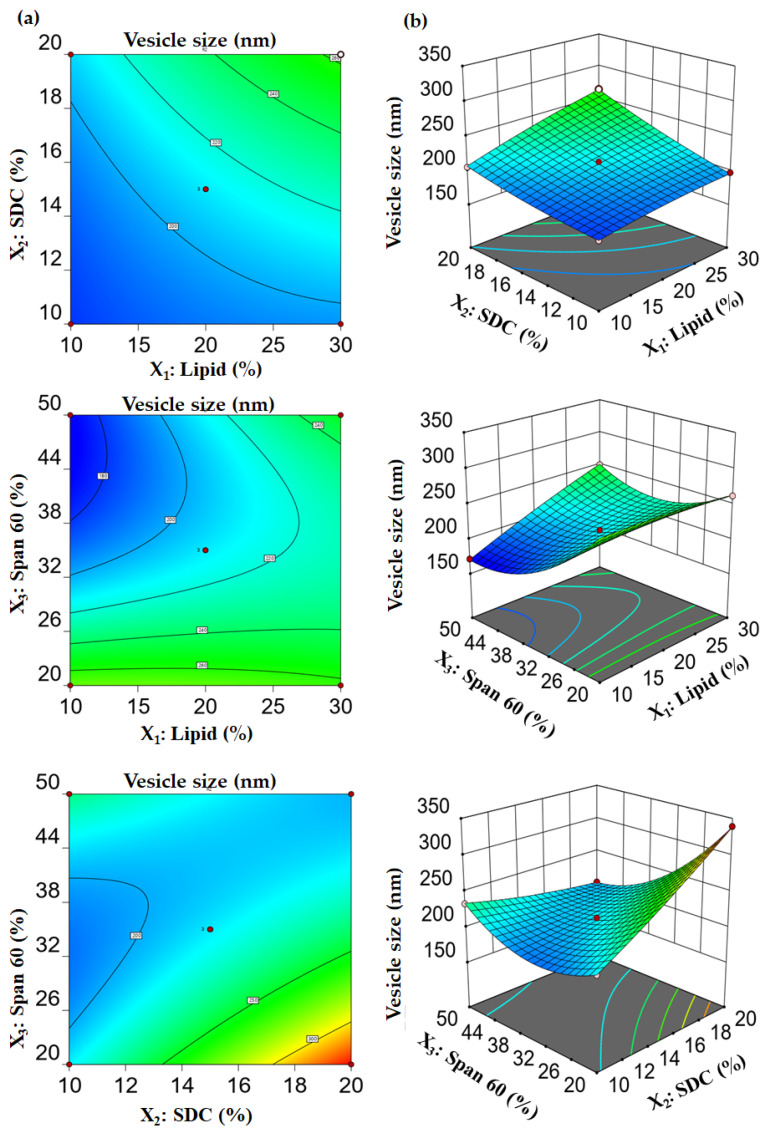
Effect of formulation factors on vesicle size (Y_2_). (**a**) Contour plots for Y_2_ and (**b**) 3D surface plots of Y_2_.

Table 1 provides a summary of the average size of vesicles in different bilosomal formulations, ranging from 171.57 ± 11.62 nm to 339.26 ± 14.64 nm. The size of the vesicles was influenced by numerous factors related to the formulation. Figure 2 demonstrates a positive correlation between lipid concentration (X_1_) and vesicle size, while keeping EA concentration (X_2_) and surfactant concentration (X_3_) constant. The average size of vesicles in bilosomes made with a lipid concentration of 10% *w*/*w* (F6; 171.57 ± 11.62 nm) was significantly smaller than those prepared with a lipid concentration of 30% (F3; 250.01 ± 9.32 nm). Under the same conditions, elevating the concentration of the edge activator (SDC) from 10% to 30% led to a significant augmentation in the size of the bilosomes. The diameter of the vesicles in F5, prepared with a 10% concentration of SDC, was 197.51 ± 12.56 nm, which was much smaller than the diameter of the vesicles in F7, prepared with a 20% concentration of SDC, which was 263.34 ± 12.12 nm. The significant enlargement of vesicle size can be attributed to a dual effect. Firstly, the bulky steroid nature of SDC may cause an increase in vesicle size [32]. Secondly, the negatively charged nature of the bile salts may enhance the repulsion between the bilayers of the vesicles, leading to a larger vesicle size [33].

Conversely, when the lipid concentration (X_1_) and EA (X_2_) were kept constant, an increase in the concentration of the surfactant (X_3_, Span 60) led to a corresponding decrease in the size of the vesicles. The bilosomes made with a surfactant concentration of 50% (F6) exhibited a reduced vesicle size (171.57 ± 11.62 nm) in comparison to the bilosomes prepared with a surfactant concentration of 20% (F2; 273.11 ± 15.22 nm). The significant reduction in particle size with increased surfactant concentration can be due to the concurrent decrease in the interfacial tension, leading to a drop in vesicle size [34].

The polynomial equation (2) expressed the cumulative impact of the formulation variables on the vesicle size (Y_2_). The ANOVA findings, summarized in (Table S1), indicate that the model was statistically significant, with an F value of 563.56 and a *p*-value less than 0.0001.

Vesicle size = 210.46 +17.89 X_1_ + 23.44 X_2_ − 28.65 X_3_ + 10.03 X_1_ X_2_ + 22.29 X_1_ X_3_ − 39.95 X_2_ X_3_ − 3.05 X_1_^2^ + 4.17 X_2_^2^ +31.86 X_3_^2^ (2)

#### 2.2.3. Determination of the Optimum Su-BLs

A mathematical optimization method was used to obtain the optimized formula that meets the required characteristics of maximizing the entrapment efficiency % while minimizing the particle size. This method involved constructing a desirability function. The bilosomal formulation, which was created using numerical optimization, consisted of 29.8% lipid, 10% SDC, and 39.72% Span 60. The desirability of this formulation was 0.769. The actual and predicted particle size and EE values for the optimum formula were calculated and adjusted to validate the optimization process. The measured particle size, encapsulation efficiency (EE %) and drug loading capacity of the optimized formulation were 211.26 ± 10.84 nm, 80.08 ± 1.88%, and 26.69 ± 0.63%, respectively. These values closely correlated with the expected values for the optimized formula, which were 209.72 ± 2.28 nm for particle size nm and 78.72 ± 0.59%, for encapsulation efficiency. The results confirmed the effectiveness of the optimization method employed to produce Su-BLs using a 3^3^ Box–Behnken design. A close agreement was found between the predicted and actual results, revealing that the model was well-fitted, as depicted graphically in Figure S1.

### 2.3. Physicochemical Characterization of the Optimized Su-BLs

#### 2.3.1. Surface Morphology and Vesicle Size

**Figure 3:**
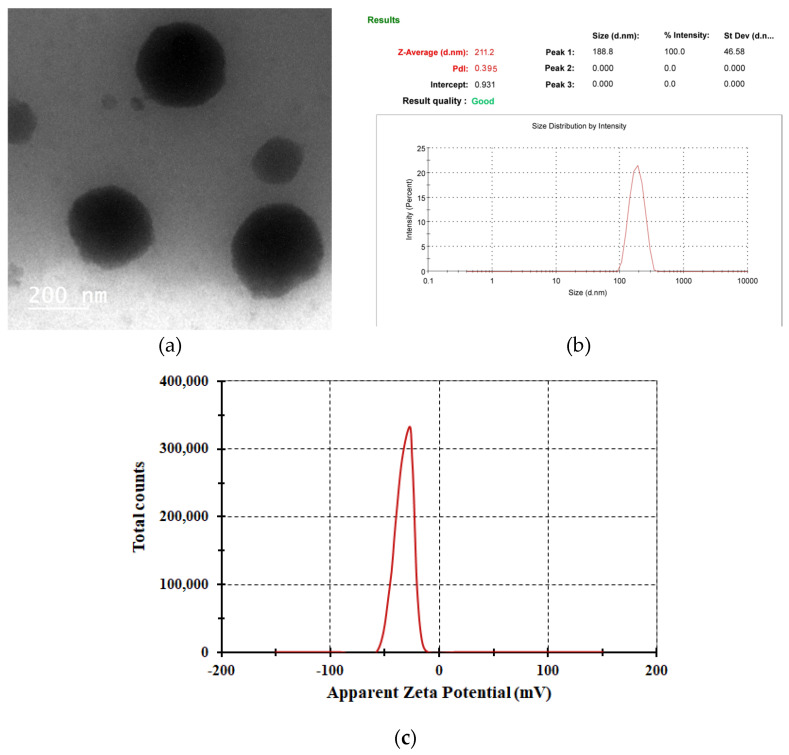
(**a**) TEM of the optimized Su-BLs and (**b**) vesicle size of the optimized Su-BLs; (**c**) zeta potential of the optimized Su-BLs.

The TEM techniques were used to evaluate the morphology of the optimized Su-BL surface. The TEM examination was utilized to determine the morphology and size of the optimized Su-loaded bilosomes. The manufactured bilosomes were clearly observed as non-aggregating spheres with smooth surfaces and distinct edges, as shown in Figure 3a. The optimized Su-BLs displayed a homogenous size distribution with an average particle size of 211.26 ± 10.84 nm with a PDI value of 0.395 (Figure 3b), demonstrating a uniform size distribution. The small size of these vesicles confirms that bilosomal vesicles are effective nano-carriers for delivering Sulpiride through the skin. Zeta potential is a measurable characteristic that may be utilized for predicting the physical stability of the vesicular formulations [35]. In our study, the zeta potential of the optimized formulation (Su-BLs) was measured to be −26.9 ± 1.4 mV, as displayed in Figure 3c. The presence of a high level of negativity suggests that the produced bilosomal vesicles had a desirable level of stability. A zeta potential greater than ±30 mV indicates strong electrostatic repulsion between particles with the same charge, which prevents them from aggregating and improves their physical stability [36].

#### 2.3.2. Differential Scanning Calorimetry

**Figure 4:**
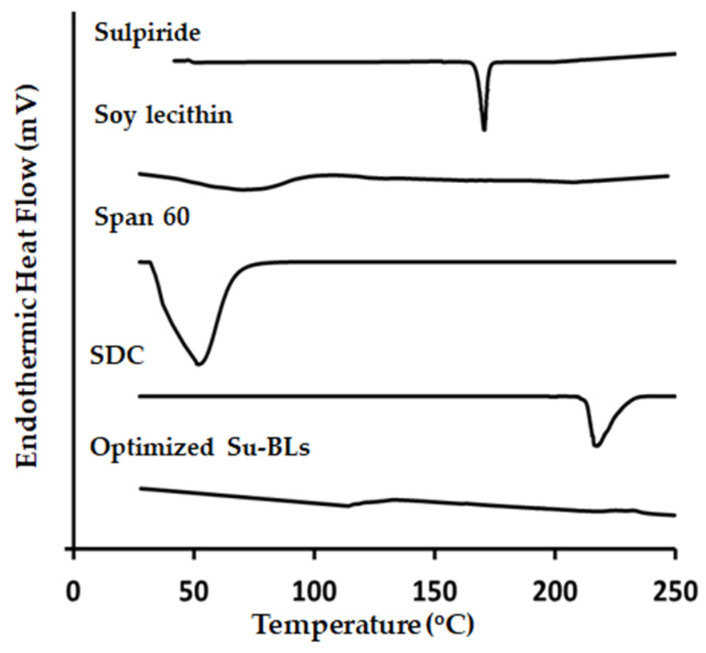
DSC thermography of Su, Soy lecithin, Span 60, SDC, and Su-BLs.

The physical properties, purity, and interactions of Su samples with excipients were evaluated using the DSC thermal analysis technique. Figure 4 displays the DSC thermograms of various substances, including pure Su, Span 60, soy lecithin, SDC, and the optimized bilosomal formulation. The DSC thermogram of pure Su showed a clear and sharp peak at 175 °C, which corresponds to its melting point. The DSC thermography of Span 60 and SDC exhibited endothermic peaks at temperatures of 52.04 °C and 217.49 °C, respectively. Conversely, the thermogram of the optimized bilosomal formula displayed no discernible sharp peaks, indicating that the encapsulated Su was incorporated into the bilosome’s vesicular bilayers well.

### 2.4. Organoleptic Evaluation and Characterization of Su-Bl Gel

The Sulpiride-loaded bilosomal gel was prepared using 4% *w*/*w* HPMC polymer. The Su-BL gel exhibited a uniform and smooth texture, without any indications of phase segregation. The pH of the Su-BL gel was measured to be 6.3 ± 0.21, which is within the allowed range for topical application [37]. This pH level ensures that the gel will not cause any irritation when applied to the surface of the skin, even with long-term use, and promotes good compatibility with the skin. The Su-BL gel that were created had viscosity values of 10.400 ± 170.59 cp, which indicates that they had a good consistency for application onto the skin. Furthermore, the Su-BL gel exhibited a favorable spreadability of 3.90 ± 0.36 cm, indicating that it rapidly spreads when applied to the skin [38].

### 2.5. In Vitro Drug Release Investigation

**Figure 5:**
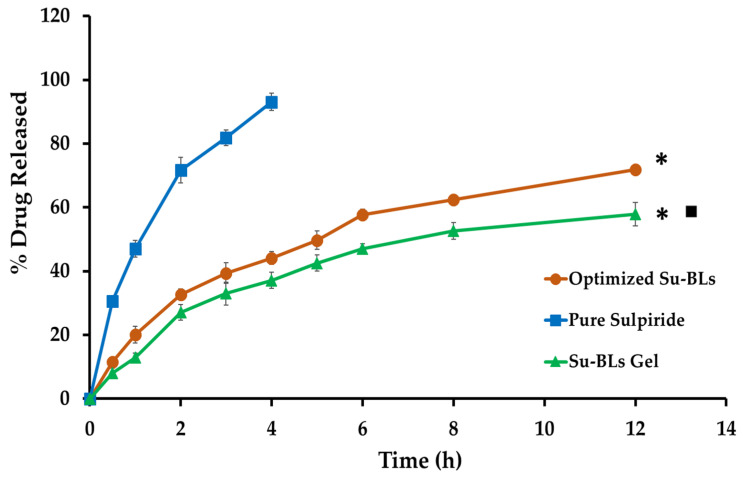
In vitro release profiles of the optimized Su-BLs, Su-BL gel, and pure Sulpiride. Results are expressed as the mean ± SD of three experiments. * *p* < 0.05 compared to pure Sulpiride; ■ *p* < 0.05 compared to Su-BL gel.

The drug release patterns from the pure Sulpiride were significantly higher than those of the tested bilosomal preparation (*p* < 0.05, as depicted in Figure 5). It was clear that about 90% or more of the drug was released from the pure drug within 4 h. On the other hand, the optimized Su bilosomal formulations exhibited biphasic release patterns, with an initial rapid release of the drug within the first three hours followed by a sustained release over twelve hours (71.88 ± 1.69%). About 40% of the drug that was encapsulated was released quickly in the first three hours. This rapid release was because of the drug, which adsorbed to the bilosomal vesicles. The drug then released more slowly because it was retained in the lipid bilayer of the bilosomes [33]. The biphasic release profile of Su from bilosomal vesicles is expected to have significant advantages. It would accelerate the onset of medication action and allow patients to maintain treatment with fewer doses through the day. Additionally, it was observed that a considerably larger amount of Sulpiride was released from bilosomes (Su-BLs) compared to bilosomal gel (Su-BLs gel), as shown in Figure 5. This observation might be attributed to the lower viscosity of Su-BLs compared to Su-BL gel, which allows the encapsulated Sulpiride to diffuse more easily into the surrounding medium.

### 2.6. Ex Vivo Permeation of Su-Bl Gel

**Figure 6:**
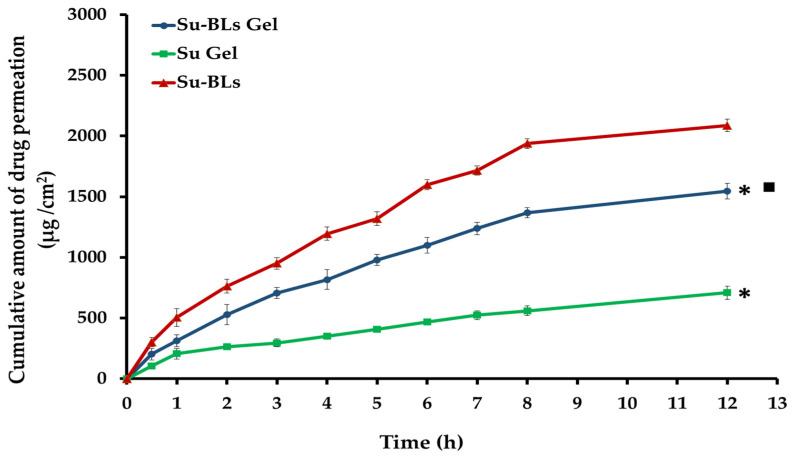
Ex vivo permeation of Su-BLs, conventional Su gel and Su-BL gel. * *p* < 0.05 compared to Su-BLs; ■ *p* < 0.05 compared to Su gel.

The in vivo performance of the Su-BL gel was predicted by conducting ex vivo permeation investigations through the skin. Figure 6 illustrates the ex vivo penetration of the Su-BL gel through the skin of the abdomen, comparing it to both Su-bilosomes (Su-BLs) and conventional Su gel. The Su-BL gel exhibited significantly higher skin permeation compared to plain Su gel (*p* < 0.05). The Su-BL gel allowed for a total permeation of 1545.44 ± 63.81 μg/cm^2^ of Sulpiride during a 12 h period, while the plain Su gel only allowed for a permeation of 708.94 ± 55.01 μg/cm^2^ of Sulpiride. In addition, the Su-BL gel exhibited a greater flux value (J_max_; 178.7 μg/cm^2^/h), whereas the plain Su gel had a J_max_ of 131.78 μg/cm^2^/h. The significant increase in bilosome flux can be attributed to two factors. Firstly, the small size and high lipid content of Su-BLs facilitate the effective permeation of bilosomes through the skin. Secondly, the bile salts (SDC) in the bilosomes act as permeation enhancers, effectively improving drug penetration by bypassing the function of the skin barrier. Notably, Su-BLs had a significantly increased ex vivo skin permeability of 2088.35 ± 52.27 μg/cm^2^ of Sulpiride over 12 h compared to Su-BL gel, which had a penetration of 1545.44 ± 63.81 μg/cm^2^. The lower penetration of Su from Su-BL gel compared to bilosomal vesicles can be attributed to the increased viscosity of the bilosomal gel compared to bilosomes, which led to a reduction in the release of the drug from Su-BL gel and subsequently lower permeability [39]. Significantly, the transdermal diffusion of Su from both Su-BLs and Su-BL gel was approximately 3.2 and 2.4 times greater than that of plain Su gel. This highlights the superior performance of Su-BLs in terms of maintaining Su release and facilitating greater penetration through the skin.

### 2.7. Pharmacokinetic Analysis

**Figure 7:**
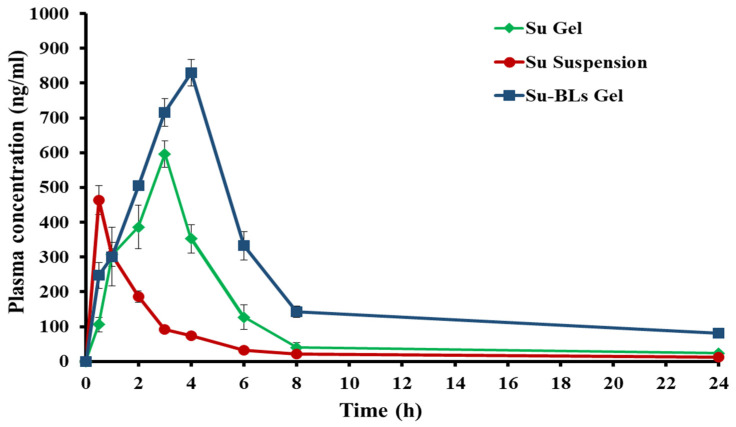
Plasma concentration versus time curve of oral Su, conventional Su gel topically, and Su-BL gel.

Figure 7 illustrates the pharmacokinetic characteristics of Su following the oral administration of a Su suspension (10 mg/mL) and the transdermal application of either conventional Su gel or Su-BL gel. Figure 7 demonstrates that both Su gel and Su-BL gel formulations resulted in elevated Su plasma concentrations, exceeding those achieved with oral Su suspension. The maximum plasma concentrations (C_max_) of Su after oral administration and after applying Su gel and Su-BL gel to the skin were 464.24 ± 58.45 ng/mL, 604.01 ± 54.68 ng/mL, and 829.56 ± 39.29 ng/mL, respectively. The comparatively lower maximum concentration (C_max_) of Su after being taken orally, in comparison to gel formulations, could be due to the limited solubility of Su in the aqueous medium, which would negatively affect its oral absorption. Conversely, the increased maximum plasma concentration (C_max_) of Su-BL gel, in comparison to conventional Su gel, can be attributed to the improved ability of the bilosomal formulation to penetrate the skin, resulting in the effective delivery of Su into the systemic circulation.

The AUC_0–24_ for the gel formulation containing Su-BLs was determined to be 5410.65 ± 559.81 ng/mL·h, which was significantly (*p* < 0.05) greater than the AUC_0–24_ values of both conventional Su gel (2603.83 ± 237.57 ng/mL·h) and oral drug suspension (1207.30 ± 94.23 ng/mL · h); see Table 2. Additionally, the Su-BL gel significantly prolonged the residence time for Su remaining in the circulatory system. The mean residence time (MRT) of the Su-BL gel was 7.03 ± 0.43 h, which was significantly greater than the MRTs of conventional Su gel or oral Su suspension (5.31 ± 0.15 h and 4.76 ± 0.22 h, respectively). The observed increase in the mean residence time (MRT) of Su-BL gel could be related to the extended and gradual release of Su from the bilosomes, as well as the reduced systemic clearance of the drug. The systemic bioavailability of Su was greatly increased by including it in a bilosomal gel. The Su-BL gel exhibited a relative bioavailability with a 4.5-fold increase compared to the oral Su suspension and an approximately 2-fold increase in the relative bioavailability compared to the conventional Su gel. The enhanced bioavailability observed after applying Su-BL gel on the skin can be ascribed to the presences of bilosomal vesicles, which served as a highly effective means of transporting Su by successfully bypassing the skin barriers [27]. These results indicate that the absorption of Su into the systemic circulation may be greatly enhanced when it is included in the bilosomal gel, compared to conventional gel and oral drug suspensions.

## 3. Conclusions

The present work introduces a novel kind of vesicle termed Sulpiride-loaded bilosomal vesicles (Su-BLs) as a potential transdermal carrier for treating psychotic disorders. Su-BLs were formulated and adjusted utilizing a 3^3^ level Box–Behnken design. The optimized Su-BL formula exhibited a nanoscale-size distribution and a satisfactory entrapment efficiency, and demonstrated effectiveness in maintaining controlled drug release in vitro for a duration of 12 h. Furthermore, the optimum Su-BL was subsequently integrated into an HPMC gel. The Su-BL gel had favorable physical characteristics and demonstrated a considerably greater transdermal permeation in comparison to the conventional Su gel. Importantly, as compared to conventional SU gel or oral Sulpiride solution, in vivo experiments showed that transdermal application of Su-BL gel significantly enhanced Su pharmacokinetics. Ultimately, bilosomes appear to be an effective vehicle for delivering Su transdermally in order to effectively treat psychosis.

## 4. Materials and Methods

### 4.1. Materials

Sulpiride was supplied by Memphis Pharmaceuticals and Chemical Industries (Cairo, Egypt). Soy lecithin, hydroxypropyl methyl cellulose, sodium deoxycholate (SDC), Span 60, chloroform, and methanol were acquired from Sigma-Aldrich (St. Louis, MO, USA).

### 4.2. Manufacturing of Sulpiride-Loaded Bilosomes (Su-BLs)

Sulpiride-loaded bilosomes (Su-BLs) were prepared using the thin film hydration process [40]. In summary, soy lecithin, cholesterol, Span 60, and Su were dissolved in a mixture of methanol and chloroform (1:1) in a round-bottomed flask. The organic phase was evaporated at 75 rpm and 60 °C, utilizing a rotatory evaporation apparatus at reduced pressure, which led to the development of a thin layer of lipids which was hydrated using ten milliliters of PBS (pH 7.4) that included sodium deoxycholate. The resulting bilosome dispersion was sonicated 3 times, each time lasting three minutes, with an interval of five minutes between each cycle. This process led to the production of bilosomes with an appropriate vesicle size. The developed bilosomes containing Su were kept at a temperature of 4 °C in a refrigerator until needed for future applications.

### 4.3. Sulpiride-Loaded Bilosome Optimization

A 3^3^ Box–Behnken design was generated utilizing Design Expert software^®^ (version 12, StatEase Inc., Minneapolis, MN, USA) to examine the impact of lipid concentration (X_1_), sodium deoxycholate concentration (X_2_), and Span 60 concentration (X_3_) as three formulation factors on the entrapment efficiency (EE%; Y_1_) and vesicle size (Y_2_) of Su-loaded bilosomes (Table 3).

The influence of the three formulation factors on the dependent variables was examined through the utilization of several experimental models, including quadratic, linear, and second order models. The ANOVA data and the regression coefficients were analyzed to choose the most suitable model. The correlation among the formulation factors and dependent variables was subsequently established through the utilization of 3D response plots and polynomial equations. Ultimately, the point prediction method, employing the desirability technique, was subsequently utilized for selecting the most optimized formula [41]. A total of fifteen runs were formulated under the conditions of our experiment (Table 1).

### 4.4. Characterization of Su BLs

#### 4.4.1. Surface Morphology, Vesicle Size, and Zeta Potential Determination

The TEM technique (JEM-2100, JEOL, Tokyo, Japan) was utilized to assess the morphology of bilosomal vesicles. The optimum bilosomes were desiccated at ambient temperature on a copper grid coated with carbon followed by analysis using a microscope at ambient temperature [42].

The size and zeta potential of the bilosomes loaded with Su were determined using a Nano ZS Zetasizer (Nano–ZS90, Malvern Instruments Ltd., Malvern, Worcestershire, UK) at a temperature of 25 ± 1.0 °C, maintaining a scattering angle of 90°. The bilosomal dispersion was diluted with deionized water to attain the necessary level of scattering intensity. Then, the vesicles were exposed to laser diffraction for vesicle size determination [43].

#### 4.4.2. Entrapment Efficiency % Determination

In summary, the bilosomes containing Su was subjected to centrifugation at a speed of 15,000 rpm at a temperature of 4 degrees Celsius for a duration of one hour using a cooling centrifuge. Next, the supernatant that remained after centrifugation was extracted, and the amount of unencapsulated drug that was not trapped was measured using a spectrophotometer (U.V. Spectrophotometer, Uviline 9100, SCHOTT-EU, Mainz, Germany) at a wavelength of λmax of 293 nm [44]. The entrapment efficiency, expressed as a percentage (% EE), was calculated using the following the following equation [45]:

$$\%EE=\frac{\mathrm{Total}\;\mathrm{Su}-\mathrm{Free}\;\mathrm{Su}}{\mathrm{Total}\;\mathrm{Su}}\times 100$$

#### 4.4.3. Drug Loading % Determination

The drug loading percentage was determined by measuring the ratio of the amount of drug entrapped to the total amount of lipid utilized in vesicle formation, as illustrated in the following calculation [46].

$$\%\mathrm{Drug}\;\mathrm{loading}=\frac{\mathrm{Total}\;\mathrm{Su}-\mathrm{Free}\;\mathrm{Su}}{\mathrm{Total}\;\mathrm{lipid}}\times 100$$

#### 4.4.4. Differential Scanning Calorimetry (DSC)

The DSC technique (DSC-60, Shimadzu, Kyoto, Japan) was utilized to analyze the thermal properties of Sulpiride, soy lecithin, Span 60, SDC, and the optimum bilosomes loaded with Su. A standard aluminum pan was used to heat 5 mg of each sample. The heating process took place within a temperature range of 10 to 300 °C, with a scanning rate of 10 °C per minute. A nitrogen purge at a flow rate of 25 mL per minute was applied during the experiment [47].

### 4.5. In Vitro Release Investigation

The diffusion technique using a cellophane dialysis bag was utilized to evaluate the in vitro release of Su from either a Sulpiride suspension or optimized Su-loaded bilosomes (each corresponding to 10 mg). Drug suspension and Su-loaded bilosomes were placed in a dialysis bag (MWCO 14,000) that had been soaked overnight in a release medium. The bag was then suspended in 100 milliliters of PBS (pH of 7.4), which served as the release medium. The medium for release was maintained at a temperature of 37 ± 1 °C and was stirred at a speed of 100 rpm for a duration of 12 h. At certain intervals, 2 mL samples of the release media were obtained and substituted with 2 mL of fresh release media to maintain the sink condition. Drug release was assessed using spectrophotometry at a wavelength of 293 nm after suitably diluting the samples [4].

### 4.6. Development of Su-Loaded Bilosome Gel (Su-BL Gel)

The optimized Su bilosomal formulation was added to a 4% *w*/*w* HPMC gel polymer [39]. In summary, a certain amount of HPMC was evenly distributed in a small quantity of distilled water, mixed well, and left undisturbed for a duration of 4–5 h. Next, the Su-BLs, consisting of 20 mg of Su, underwent centrifugation; the resulting pellets were then mixed into the gel base using a magnetic stirrer, resulting in a smooth gel (2% *w*/*w*) completely devoid of any aggregations. A gel containing Sulpiride was made in a similar manner, but instead of using a bilosomal dispersion, the plain drug was used.

### 4.7. Organoleptic Evaluation and Characterization of Su-BL Gel

The prepared gel was visually inspected in order to assess various physical parameters, including phase separation, color, clarity, and homogeneity [48]. The viscosity of the generated Su-BL gel was determined using a Brookfield-R viscometer (Model DV-II, Middleboro, MA, USA). The equipment was set to rotate at a speed of ten rpm at a temperature of 37 ± 1 degrees Celsius. The pH of the Su-BL gel was determined using a pH meter (PCT-407 Portable pH Meter, Taipei City, Taiwan). In summary, one gram of the gel was diluted with distilled water at a ratio of 1:10, and the pH was measured three times. The spreadability of the bilosomal gel loaded with Su was assessed by inserting half grams of the gel between two slides of glass, and the top slide then had a fixed weight applied to it for a duration of one minute. The spreading area’s diameter was determined for assessing the spreadability [49].

### 4.8. In Vitro Drug Release Investigation from Su-BL Gel

As mentioned earlier in Section 4.5, the same approach was employed to evaluate the rate at which Sulpiride is released from the bilosomal gel formulation, comparing it to the release rate of the pure Sulpiride and Su-BL preparation. At certain time intervals (0.5, 1, 2, 3 4, 5, 6, 8, and 12 h), 2 mL samples were removed and replaced with a new buffer. The drug content of the samples was evaluated spectroscopically at a wavelength of maximum absorption of 293 nm [50].

### 4.9. Ex Vivo Permeation Investigation

The ex vivo permeability of Su from Su-BLs, control Su gel, and optimized Su-loaded bilosomal gel was evaluated using hairless rat skin and a locally fabricated diffusion cell. The skin of the abdomen region of the rats was cleaned with phosphate-buffered saline (pH 7.4) to remove any adipose tissue or other fatty tissues before the beginning of the experiment. Subsequently, skin samples were positioned between the diffusion cell’s donor and receptor compartments so that the dermal layer was subjected to the receptor media and the stratum corneum came into contact with the donor compartment. A certain quantity of Su-BLs, Su gel, or Su-BL gel was introduced into the donor compartment. The receptor media consisted of PBS (pH 7.4) maintained at a temperature of 37 ± 1 °C and agitated at a speed of 100 rpm. At scheduled times during 12 h, 2 mL samples were extracted from the receptor media and replaced with an equivalent fresh solution. The concentration of Sulpiride in each sample was determined using spectrophotometry at a wavelength of 293 nm. The quantities of Su that penetrated through the skin of rats per unit area (μg/cm^2^) were shown against time (h). The permeation characteristics, such as the flux (J_max_) in μg/cm^2^/h and the enhancement ratio (ER), were evaluated for Su gel and Su-Su-BL gel according to the following equations [33].

$$\mathrm{Flux}=\frac{\mathrm{Amount}\;\mathrm{of}\;\mathrm{permeated}\;\mathrm{drug}}{\mathrm{area}\;\mathrm{of}\;\mathrm{permeation}\times \mathrm{time}}$$

Enhancement Ratio=(Flux from test)/(Flux from control)

### 4.10. In Vivo Experiment

#### 4.10.1. Animals

Adult male albino rats weighing between 220 and 250 g were kept in an environment that was monitored with regulated temperature and humidity. They were provided with unrestricted access to laboratory water and chow. The Ethical Committee of the University of Ha’il, KSA, approved and examined all animal experiments conducted (H-2023-361 on 19 September 2023).

#### 4.10.2. In Vivo Pharmacokinetics

The rats were divided into three groups (*n* = 5). A Sulpiride suspension (15 mg/kg) was administered orally using oral gavage to the first group. The topical application of Su gel and Su-BL gel (15 mg Su/kg) was administered to the other two groups at predetermined time intervals throughout a 24 h period. The samples of blood were obtained from the lateral tail vein and centrifuged at 5000 rpm for fifteen minutes in tubes containing heparin to collect plasma [51]. The collected plasma was frozen at a temperature of −20 °C for subsequent analysis. HPLC analysis was used to evaluate the concentration of Su in plasma samples, utilizing a 2690 Alliance HPLC system (Markham, ON, Canada) equipped with a Waters 996 photodiode array detector.

In brief, 100 microliters of plasma were combined with 50 microliters of 1 M NaOH and 5 μL of paracetamol (100 μg/mL), which served as an internal standard. The mixture was then vortexed for 10 s. The mixture was extracted with 4 mL of dichloromethane/ethyl acetate mixture (1:3, *v*/*v*) through vortex-mixing for an additional one minute at a high speed, followed by centrifugation at 5000 rpm for 10 min. The organic layer was evaporated until completely dry. Subsequently, the remaining substances were combined with 1 mL of the mobile phase, and 100 μL of the mixture was introduced into the HPLC column (Kromasil C18 (250 × 4.6 mm, 5 μm)) to measure the Su plasma concentration [4]. The mobile phase was produced by dissolving one gram of hexane sulfonic acid in 900 mL of water, followed by agitation. The volume was then adjusted to one liter with water and the pH was set to three, then blended with acetonitrile in a ratio of 80:20, volume to volume. The HPLC analysis was performed at an ambient temperature with a flow rate of 1 mL/min at a wavelength of 254 nm, using an injection volume of 100 μL. Sulpiride pharmacokinetic parameters were calculated using the PKSolver 2.0 program, including peak plasma concentration C_max_, T_max_, t_1/2_, MRT, and AUC_0–24_.

### 4.11. Analysis of Data Using Statistical Methods

One-way analysis of variance (ANOVA) was utilized to assess the significance of differences. The data were reported as mean ± standard deviation (S.D.). The statistical significance was assessed using a significance level of *p* < 0.05.

## Figures and Tables

**Table 1:** The BBD of Su-BLs’ measured responses, independent factors, and experimental runs.

Formula	X_1_	X_2_	X_3_	Y_1_	Y_2_
F1	20	15	35	56.75 ± 2.09	209.69 ± 13.01
F2	10	15	20	68.81 ± 1.49	273.11 ± 15.22
F3	30	15	50	88.29 ± 3.01	250.01 ± 09.32
F4	20	20	50	60.36 ± 3.07	201.71 ± 11.39
F5	30	10	35	75.19 ± 2.57	197.51 ± 12.56
F6	10	15	50	47.42 ± 1.38	171.57 ± 11.62
F7	30	20	35	68.96 ± 1.91	263.34 ± 12.12
F8	10	20	35	50.30 ± 2.48	205.59 ± 10.56
F9	20	15	35	57.41 ± 0.91	208.12 ± 10.24
F10	20	15	35	56.82 ± 0.69	213.58 ± 13.24
F11	30	15	20	60.49 ± 2.67	262.39 ± 09.67
F12	20	10	50	62.47 ± 1.31	233.63 ± 10.73
F13	20	20	20	52.12 ± 2.64	339.26 ± 14.64
F14	10	10	35	58.15 ± 2.07	179.87 ± 07.56
F15	20	10	20	65.23 ± 3.02	211.37 ± 11.23

X_1_—lipid concentration (%); X_2_—SDC concentration (%); X_3_—surfactant concentration (%); Y_1_, EE (%); Y_2_, particle size (nm).

**Table 2:** Pharmacokinetic characteristics of Su preparations.

Pharmacokinetic Parameter	Oral Su Suspension	Su-Gel	Su-BL Gel
C_max_ (ng/mL)	464.24 ± 58.45	604.01 ± 54.68	829.56 ± 39.29
T_max_ (h)	0.5	3	4
K_el_ (h^−1^)	0.14 ± 0.02	0.11 ± 0.01	0.075 ± 0.01
t_1/2_ (h)	5.09 ± 0.22	6.02 ± 0.26	9.311.15
AUC_0–24h_ (ng/mL·h)	1207.30 ± 94.23	2603.83 ± 237.57	5410.65 ± 559.81
MRT (h)	4.76 ± 0.22	5.31 ± 0.15	7.03 ± 0.43

**Table 3:** Box–Behnken design characteristics and experimental conditions.

Formulation Factors	Level		
	Low (−1)	Medium (0)	High (+1)
X_1_: Lipid concentration (% *w*/*w*)	10	25	30
X_2_: Edge activator (SDC) concentration (% *w*/*w*)	10	15	20
X_3_: Surfactant concentration, Span 60 (% *w*/*w*)	20	35	50
Dependent variables	Desirability constrains		
Y_1_: EE (%)	Maximize		
Y_2_: Vesicle size (nm)	Minimize		

## Data Availability

The original contributions presented in the study are included in the article, further inquiries can be directed to the corresponding authors.

## Supplementary Materials

The following supporting information can be downloaded at: https://www.mdpi.com/article/10.3390/gels10040239/s1, Table S1: Results of the statistical analysis of all dependent variables Y_1_ and Y_2_, Figure S1: Linear correlation plots (a,b) between predicted and actual values and (c,d) the corresponding residual plots for different responses.
