# Supplementary material for: The Exploitation of Sodium Deoxycholate-Stabilized Nano-Vesicular Gel for Ameliorating the Antipsychotic Efficiency of Sulpiride
Source: Gels. 2024 Mar 31;10(4):239. doi: 10.3390/gels10040239 (PMC11048809; doi:10.3390/gels10040239)
Supplement: Supplementary file 1 [file gels-10-00239-s001.zip › gels-2926421-supplementary.pdf]

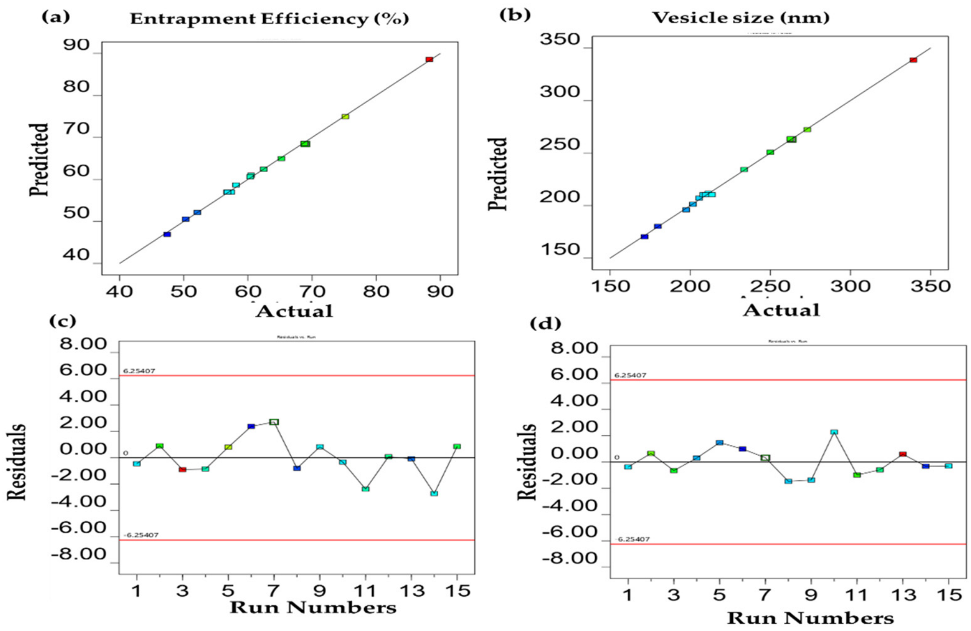

Figure S1. Linear correlation plots (**a**,**b**) between predicted and actual values and (**c**,**d**) the corresponding residual plots for different responses.

Table S1. Results of statistical analysis of all dependent variables Y_1 and Y_2.

Source	Y_1		Y_2	
	**F-value**	***p*-value**	**F-value**	***p*-value**
Model	480.16	< 0.0001*	563.56	< 0.0001*
X_1: Lipid concentration (%)	1665.44	< 0.0001*	496.11	< 0.0001*
X_2: Edge activator, SDC (%)	306.81	< 0.0001*	851.82	< 0.0001*
X_3: Surfactant concentration, Span 60 (%)	50.58	0.0009*	1272.61	< 0.0001*
Lack of Fit	3.81	0.2150	0.4224	0.7584
R^2 analysis				
R^2	0.9988		0.9990	
Adjusted R^2	0.9968		0.9972	
Predicted R^2	0.9839		0.9925	
Adequate Precision	86.2734		90.6236	

X_1- Lipid concentration (%); X_2- edge activator (SDC) concentration (%); X_3- surfactant concentration (%); Y_1, EE (%); Y_2, particle size (nm); *, significant.
